# Omega-3 and -6 Fatty Acids Alter the Membrane Lipid Composition and Vesicle Size to Regulate Exocytosis and Storage of Catecholamines

**DOI:** 10.1021/acschemneuro.3c00741

**Published:** 2024-02-12

**Authors:** Chaoyi Gu, Mai H. Philipsen, Andrew G. Ewing

**Affiliations:** Department of Chemistry and Molecular Biology, University of Gothenburg, 41390 Gothenburg, Sweden

**Keywords:** alpha-linolenic acid, linolenic acid, catecholamine, exocytosis, vesicular content, phospholipids

## Abstract

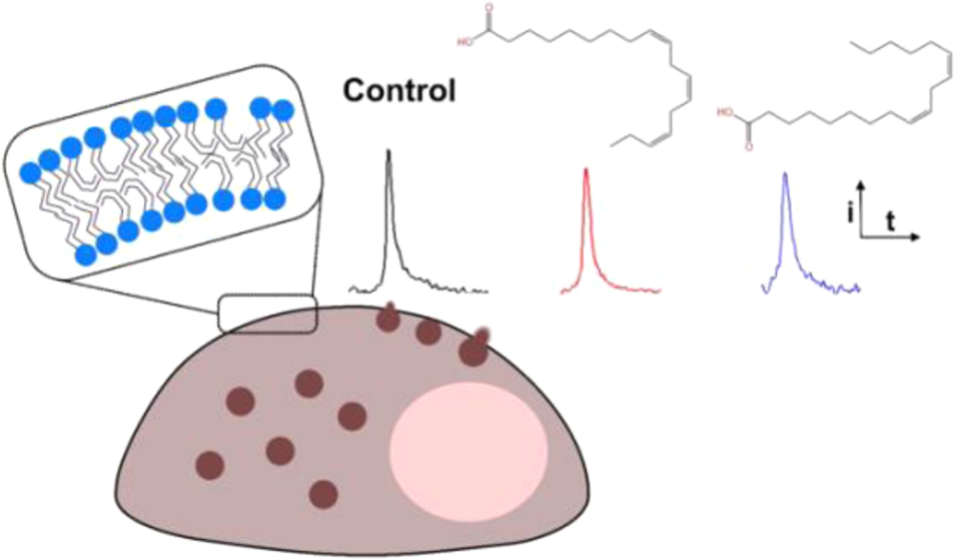

The two essential fatty acids, alpha-linolenic acid and linoleic acid, and the higher unsaturated fatty acids synthesized from them are critical for the development and maintenance of normal brain functions. Deficiencies of these fatty acids have been shown to cause damage to the neuronal development, cognition, and locomotor function. We combined electrochemistry and imaging techniques to examine the effects of the two essential fatty acids on catecholamine release dynamics and the vesicle content as well as on the cell membrane phospholipid composition to understand how they impact exocytosis and by extension neurotransmission at the single-cell level. Incubation of either of the two fatty acids reduces the size of secretory vesicles and enables the incorporation of more double bonds into the cell membrane structure, resulting in higher membrane flexibility. This subsequently affects proteins regulating the dynamics of the exocytotic fusion pore and thereby affects exocytosis. Our data suggest a possible pathway whereby the two essential fatty acids affect the membrane structure to impact exocytosis and provide a potential treatment for diseases and impairments related to catecholamine signaling.

## 1 Introduction

Alpha-linolenic acid (ALA) and linoleic acid (LA) are the two fatty acids (FAs) that cannot be synthesized by the human body and are thus considered to be the two essential FAs. ALA belongs to the omega-3 FA family and has a long carbon chain with 18 carbons and three *cis* double bonds. LA, on the other hand, belongs to the omega-6 FA family and has 18 carbons and two *cis* double bonds on its carbon chain. Both ALA and LA function as precursors in the synthesis of a series of critical omega-3 and omega-6 polyunsaturated fatty acids (PUFAs) via a few desaturation and elongation reactions,^1^ but the efficiency of synthesis drops significantly along the reaction steps.^2^ Eicosapentaenoic acid and docosahexaenoic acid (DHA) are converted from ALA and have significant impacts on various cellular functions, inflammatory processes, certain disorders, and cognition.^3^ DHA, in particular, is highly abundant in the brain and is not only critical for neuronal development and brain growth in infants but also necessary for maintaining adult brain functions.^4^ The lack of DHA or other omega-3 PUFAs in the brain has been shown to cause a negative impact on cognition and alter dopaminergic neurotransmission, which leads to abnormal locomotor activity.^5,6^ Arachidonic acid (AA), which is synthesized from LA, is as important as DHA for maintaining neurological health. Moreover, its neuroprotective roles and the involvement of AA in repairing neurons have also been demonstrated.^7^ As ALA, LA, and their FA turnover products can be incorporated into cell membranes upon uptake,^8^ relating this to their effects on transmission on the single-cell level is of importance to understand the effects of these FAs. Additionally, this may provide pathways for catecholamine release and storage related to inflammation and oxidative stress in the brain and treatments for disorders linked to abnormal activity of catecholamine neurons.

Neurotransmission typically occurs via a process called exocytosis, during which neurotransmitter-encapsulated secretory vesicles fuse with the cell membrane to release the transmitter cargo to the extracellular environment. The released transmitters can then interact with one or a few adjacent cells to allow for information passage within a neural network. The fusion process during exocytosis is energy-driven as both cellular and vesicular membranes are intrinsically stable. Thus, the membranes must overcome an energy barrier to fuse, and redistribution of membrane phospholipids can facilitate this process.^9^ Regions on the membrane where fusion takes place possess higher curvature than other areas, meaning that lipid species with higher curvature will migrate into fusion regions, while the ones with lower curvature will be prevalently found in flatter regions. Lipid rearrangement continues when the fusion process persists.^10^ Several studies have shown that exogenously supplied phospholipids are capable of altering exocytosis, both the amount of release and the activity of the fusion pore, supporting the importance of lipids in neurotransmission.^11,12^ However, as phospholipids are made up of a headgroup and two FA tails, affecting different properties, e.g., headgroup species, number of carbons, saturation degree, etc., alters the membrane structure differently and may lead to differential effects on exocytosis. For example, a higher number of carbons makes the membrane more rigid, but this can be compensated by increased unsaturation.^13^ Therefore, a more thorough understanding of how lipids are involved in neurotransmission is needed.

We combined two electrochemical techniques, namely, single cell amperometry (SCA) and intracellular vesicle impact electrochemical cytometry (IVIEC), to study exocytosis and the vesicular content. Both techniques have a sufficient temporal resolution, down to the submillisecond time scale, to resolve events from individual vesicles. SCA was developed in the 1990s by the Wightman group to measure exocytosis of electroactive neurotransmitters, e.g., catecholamines.^14^ It not only allows the quantification of the number of molecules from individual vesicular release events but also offers dynamic information in terms of the exocytotic fusion pore, which helps uncover the mechanisms of exocytosis.^15^ IVIEC, developed in 2015, enables direct quantification of the total neurotransmitter content within individual vesicles inside single cells.^16^ The combination of the two techniques shows partial release as the dominant mode of exocytosis.^17−19^ Transmission electron microscopy (TEM) is a technique applied to visualize subcellular ultrastructures. Large dense-core vesicles (LDCVs) are one type of secretory vesicles that enclose a core of protein aggregates, which binds neurotransmitters and other small molecules.^20^ This dense-core structure appears to be darker under TEM than the area surrounding it, thus making TEM a popular method to study the alteration of dense-core properties. To examine changes of membrane lipid species, time-of-flight secondary ion mass spectrometry (ToF-SIMS) imaging was used with advantages of a high spatial resolution and the ability to analyze high-mass molecules like intact lipids. In addition to that, ToF-SIMS in general is surface-sensitive, allowing identification and localization of biomolecules across the surface of the cell membrane as well as enabling relative quantification. These together have made it a powerful tool for biological and medical research.^21−23^

In this paper, we combined electrochemical and imaging techniques, including SCA, IVIEC, TEM, and ToF-SIMS with the J105 chemical imager, to investigate the effects of ALA and LA, the two essential FAs, on vesicular release and the vesicular transmitter content via the alteration of membrane phospholipids. Pheochromocytoma (PC12) cells are a model cell line established in 1976 and have since then become a useful model for exocytosis studies, e.g., neural secretion and differentiation.^24,25^ We used PC12 cells here as they possess LDCVs that secrete catecholamine transmitters, mainly dopamine, in response to the elevation of intracellular calcium. Moreover, the ability to store and release transmitters, including dopamine, makes PC12 cells attractive for the study of ALA and LA deficiencies and how their corresponding derivatives impair transmission. SCA and IVIEC were applied to measure transmitter release and storage, respectively. We observed decreases in both the amount of release and the amount of the transmitter content due to either ALA or LA incubation. Notably, the changes are more significant with ALA. The dynamics of exocytosis revealed by the peak analysis of SCA indicates that the duration of the exocytotic fusion pore is significantly shortened upon ALA incubation. The size of LDCVs as visualized by TEM is reduced due to ALA or LA, matching the decreased transmitter content quantified by IVIEC. LA induces even smaller-sized vesicles than ALA does, meaning that the vesicular catecholamine concentration is higher in LA-incubated cells. Results obtained from ToF-SIMS suggest that both FA incubations lead to an elevated number of double bonds within membrane phospholipids, making cell membranes less rigid and more flexible. The activity of proteins such as dynamin is thus altered by the membrane flexibility to regulate fusion pore dynamics. The amount of lipid turnover into membranes and the location of their incorporation upon FA incubation can also contribute to the observed alteration of neurotransmission. Our data show a correlation between alteration in the membrane structure and change in chemical transmission via exocytosis and provide a possible mechanism regarding the two essential FAs in diseases and impairments related to catecholamine function.

## 2 Results and Discussion

### 2.1 Both ALA and LA Reduce the Average Amount of Exocytotic Catecholamine Release But Do Not Change the Number of Release Events

**Figure 1:**
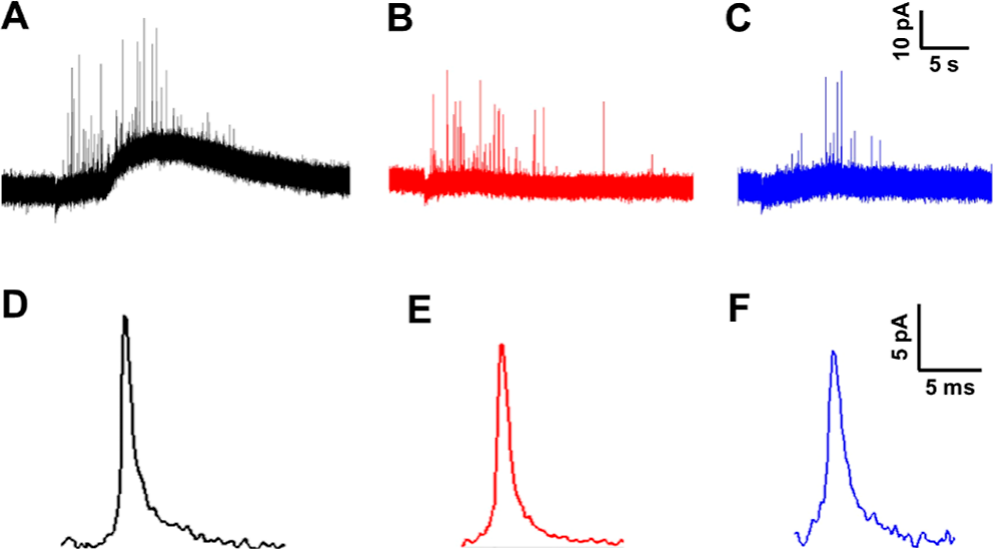
Representative amperometric traces (A–C) and corresponding average spike shape (D–F) of exocytosis from PC12 cells without FA treatment (A,D), with 24 h of 100 μM ALA treatment (B,E), and with 24 h of 100 μM LA treatment (C,F).

PC12 cells were incubated with 100 μM either ALA or LA for 24 h before SCA was applied to measure the response to exocytosis. Nanotip electrodes were used to perform SCA in this study to offer a better comparison to the vesicular content quantified by IVIEC with the same type of electrodes.^16,26^ Exocytotic release was chemically triggered by applying a stimulation solution containing a higher concentration of K^+^ ions than the bathing solution surrounding the cell, and the duration of each stimulation was 5 s. In response to this, vesicles fuse transiently with the cell membrane to release catecholamines. By placing a nanotip electrode in close contact to the cell membrane and applying a constant +700 mV potential, the released transmitters that are electroactive, e.g., catecholamine, are oxidized on the electrode surface, and exocytosis from many vesicles within a single cell is measured as an amperometric trace containing a cluster of spikes. Figure 1A–C shows examples of amperometric traces obtained from the control, 100 μM ALA-treated, and 100 μM LA-treated PC12 cells, respectively, and Figure 1D–F illustrates the average spike shapes of the corresponding treatment groups. Both ALA and LA incubations give rise to smaller amperometric spikes than those of the control cells (Figure 1D–F). In addition, they also led to the observation of decreased frequency of exocytotic spikes in comparison to that in the control group (Figure 1A–C, the average number of exocytotic release events per cell is shown as Figure S1). However, due to the electrode fabrication method (details are included in the Methods section), surface area varies among nanotip electrodes, which makes it difficult to compare exocytotic frequency quantitatively.

**Figure 2:**
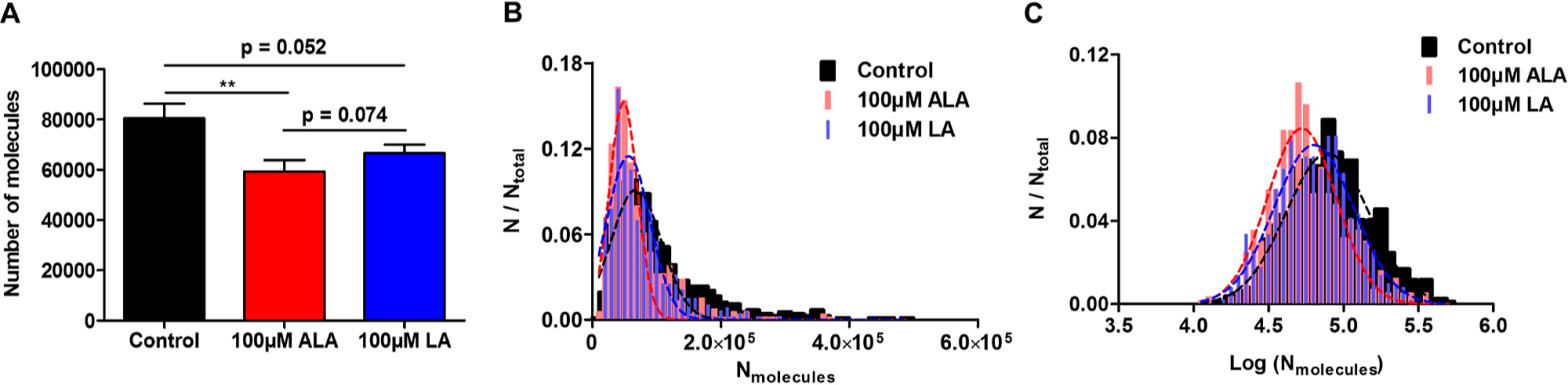
(A) Average number of molecules released per exocytotic event from PC12 cells without or with FA treatment. Error bars represent the SEM. Data sets were compared with a two-tailed Mann–Whitney rank-sum test, **p < 0.01, and other *p* values are shown in the graph. (B) Normalized frequency histograms describing the distribution of molecules and (C) log (molecules) released from control PC12 cells (725 events from 25 cells), 24 h 100 μM ALA-treated cells (527 events from 21 cells), and 24 h 100 μM LA-treated cells (476 events from 21 cells). Bin size = 1 × 10^4^ molecules for (B) and 0.05 for (C). Data were fitted to a Gaussian distribution.

To investigate the alteration of exocytotic release caused by ALA or LA incubation, the area under each amperometric spike was integrated, and by applying Faraday’s law (*N* = *Q*/*nF*), the number of molecules released during an individual release event was calculated. In Faraday’s law, the time integral of each spike is expressed as *Q*, *n* is the number of electrons transferred during the oxidation reaction which is 2 for the oxidation of catecholamine molecules, and *F* is the Faraday constant. A significantly lowered average release amount is caused by ALA incubation, as shown in Figure 2A, while LA also triggers a decline in average exocytotic release, but not as large as ALA does. The distribution of the number of molecules as well as the log number of molecules from all exocytotic release events measured by SCA was examined and compared among the three groups. It can be observed from Figure 2B,C that treatment with either ALA or LA shifts the molecule distribution toward a fewer number of molecules. To be more specific, for Figure 2B, the medians of molecular distribution are 80,300, 55,700, and 61,900 for the control, ALA-treated, and LA-treated groups, respectively. When comparing between the two treatment groups, the median of molecular distribution for ALA is lower than that for LA, indicating that compared to LA, ALA decreases a higher amount of exocytotic release within the entire vesicle population, and this shows the same trend as what has been observed with average exocytotic release per cell (Figure 2A).

**Figure 3:**
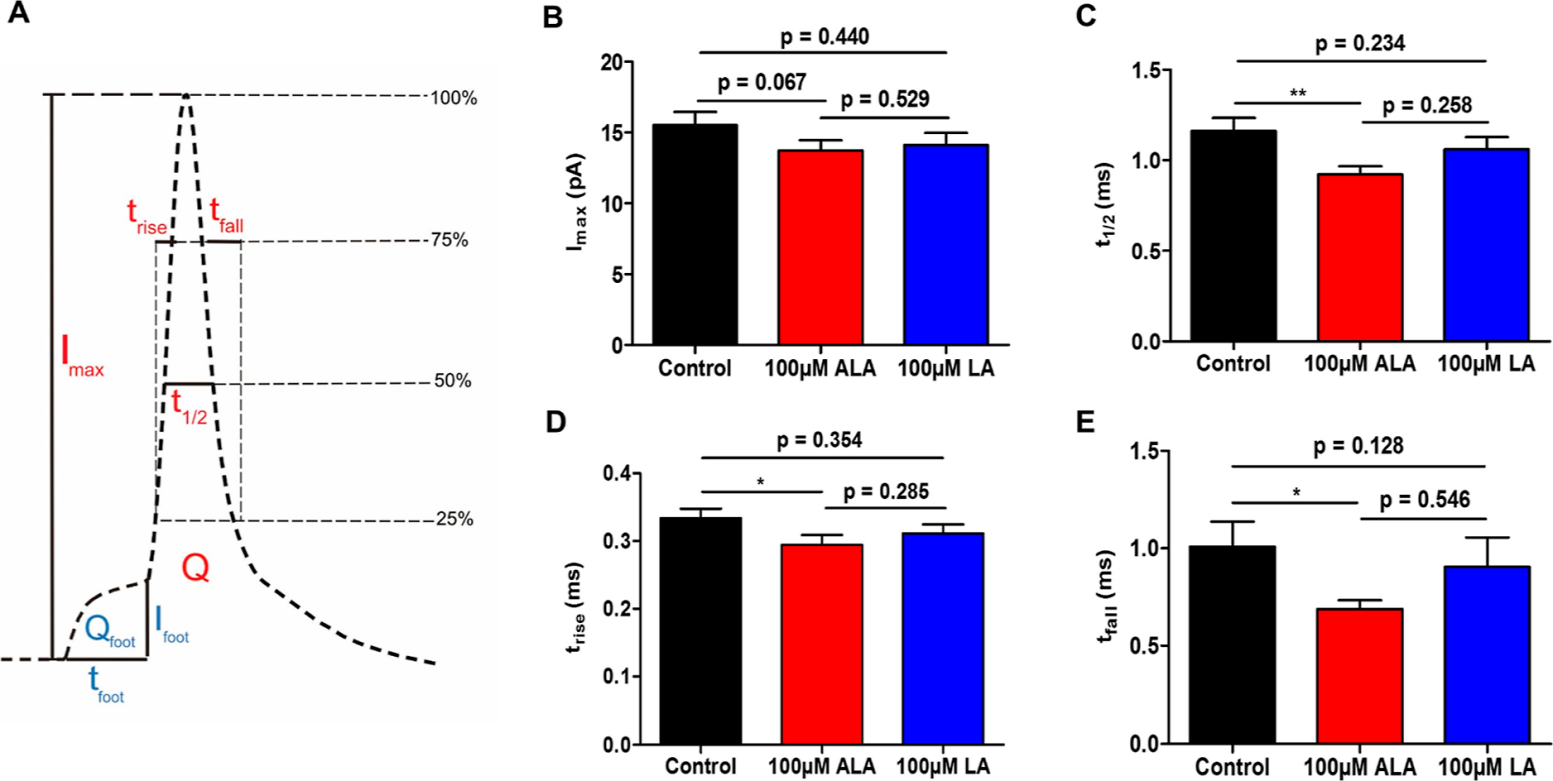
(A) Scheme illustrating the different parameters used to analyze an amperometric spike. Comparison of spike parameters regarding the main spike including (B) spike current *I*_max_, (C) half spike width *t*_1/2_, (D) rise time *t*_rise_, and (E) fall time *t*_fall_ measured by SCA from the control (25 cells), 24 h 100 μM ALA treatment (21 cells), and 24 h 100 μM LA treatment (21 cells) cells. Comparison of parameters regarding the prespike foot including *I*_foot_, *t*_foot_, and *Q*_foot_ can be found in Figure S2 in the Supporting Information. Error bars represent the SEM. Data sets were compared with a two-tailed Mann–Whitney rank-sum test, *p < 0.05, **p < 0.01, and other *p* values are shown in the graph.

The parameters of amperometric spikes obtained by SCA can be further analyzed to add dynamic information about the exocytotic release events. As illustrated in Figure 3A, *I*_max_ is the current at the maximum height of the main spike, *t*_1/2_ is the width of the main spike at its half height, *t*_rise_ is the time taken to rise from 25 to 75% of the main spike height, and *t*_fall_ is the time taken to fall from 75 to 25% of the main spike height. For exocytosis to occur, a transient fusion pore must be formed between the vesicle lumen and the cell membrane to allow dissipation of neurotransmitters to the extracellular environment. The fusion pore begins with a relatively small size and then continues to expand, during which the efflux of transmitters is measured and shown as the rising phase of the amperometric main spike. The final destiny of the fusion pore can be either expanding to the maximum degree to completely merge the vesicle into the cell membrane (full fusion) or constraining to close to allow the vesicle to leave the cell membrane (partial fusion), showing as the falling phase of the amperometric main spike.^15,27,28^ Therefore, the three time-related parameters, *t*_1/2_, *t*_rise_, and *t*_fall_, can be used to understand important properties of the fusion pore, with *t*_1/2_ representing the duration of the fusion pore, *t*_rise_ representing the time for the fusion pore to open and expand, and *t*_fall_ representing the time for transmitter diffusion in the case of full fusion and the time for both transmitter diffusion and the fusion pore to close in the case of partial fusion. *I*_max_ represents the maximum efflux of transmitters through the fusion pore and might also give a hint of the size of the fusion pore since transmitter flux is related to the pore size. As shown in Figure 3B, neither ALA nor LA treatment leads to a significant decrease regarding *I*_max_. The effect of ALA on exocytosis is mainly due to the change of the time-related parameters as ALA causes the fusion pore to expand and close significantly faster than that in the control group (Figure 3D,E). The duration of the fusion pore is thus significantly shorter upon ALA treatment, as can be seen in Figure 3C. LA treatment shortens all three time-related parameters as well but not significantly. Details regarding the four spike parameters and the comparison of them among the three groups are included as Table S1.

Previous studies have suggested that some long-chain PUFAs, which are synthesized from either ALA or LA, are capable of upregulating syntaxin protein to promote the formation of the soluble NSF attachment receptor (SNARE) complex.^29,30^ The SNARE protein family is known to be critical for several steps of exocytosis, including priming and fusion, and thus, an easier-formed SNARE complex results in an increased possibility of exocytosis or a higher frequency of exocytotic release events. In our study, we observed that neither ALA nor LA significantly affects the average number of exocytotic release events per cell (Figure S1). In addition, as the opening of the fusion pore requires full zippering of the SNARE proteins, easier-assembled SNARE complexes may help the fusion pore open faster, and this correlates with the decreased *t*_rise_ observed for ALA incubation (Figure 3D).^31^ Upon fusion, the expansion and constriction of the fusion pore are governed by a few other proteins, such as actin and dynamin.^27,28,32,33^ The interplay of these proteins determines the dynamics and stability of the fusion pore. As ALA incubation significantly shortens the duration of the fusion pore, this indicates that ALA induces an alteration of the protein interplay to accelerate the opening process of the fusion pore as well as to make the fusion pore less stable.

At the beginning of the rising phase on the amperometric main spike, a small shoulderlike increase of current is present from time to time. This corresponds to the opening of the fusion pore to a relatively small size and is typically called the “prespike foot”.^34−36^ The continuous expansion of this small fusion pore leads to the main exocytotic event. There are three parameters used to understand the feature of the prespike foot, including *I*_foot_, *t*_foot_, and *Q*_foot_, which are illustrated in Figure 3A. *I*_foot_ is the maximum current of the foot and represents the maximum transmitter efflux through the foot and perhaps the size of this small fusion pore. *t*_foot_ is the total width of the foot and represents the duration of this small fusion pore. *Q*_foot_ is the area under the foot, and the number of molecules released during the foot can be calculated from *Q*_foot_ using Faraday’s law. The result of prespike foot analysis without or with FA treatment is presented in Figure S2. There are some differences when comparing the prespike foot parameters to the parameters of the main spike. ALA treatment gives rise to a significant decrease in *I*_foot_ (Figure S2A), meaning that the maximum flux of transmitters is reduced, which is similar to what occurs to *I*_max_ except that the change of *I*_max_ is insignificant. No significant change occurs to *t*_foot_ upon ALA treatment (Figure S2B), demonstrating that the duration of the small fusion pore stays almost the same as that of the control. We did observe that the *t*_1/2_ and the *t*_rise_ of the main spike were significantly smaller; however, it is possible that the duration of the foot is not as greatly affected as the duration of the main exocytotic event. These together result in a decreased amount of molecules being released through the foot upon ALA treatment (Figure S2C), and this is consistent with the release observed for the main event. LA treatment, however, shows no significant alterations relative to the control but gives higher values relative to ALA treatment regarding the *I*_foot_ and the number of molecules released through the foot.

### 2.2 ALA and LA Decrease the Vesicle Size and, Thus, Vesicular Catecholamine Storage to Alter Exocytotic Release Dynamics

**Figure 4:**
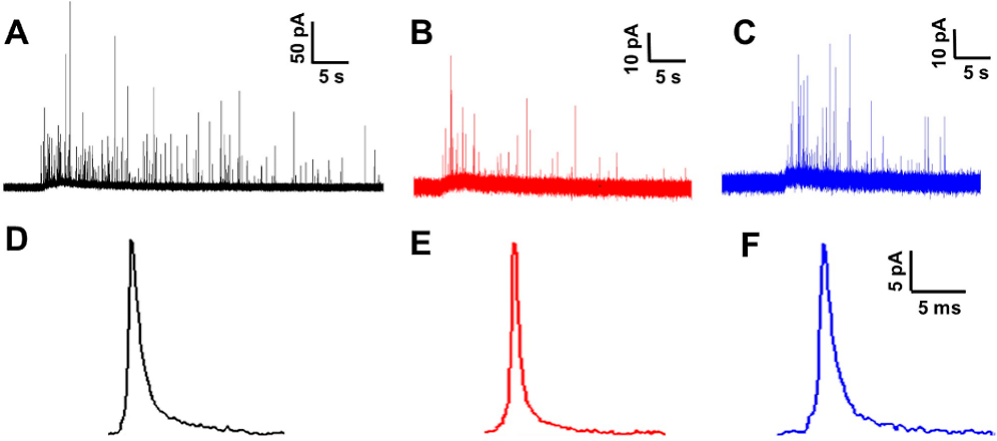
Representative amperometric traces (A–C) and corresponding average spike shape (D–F) of the vesicular content from PC12 cells without FA treatment (A,D), with 24 h of 100 μM ALA treatment (B,E), and with 24 h of 100 μM LA treatment (C,F).

To quantify the amount of catecholamine stored inside vesicles, we used IVIEC.^16^ Nanotip electrodes were employed to perform IVIEC, during which the sharp tip of the electrode was carefully pushed through the cell membrane into the cytoplasm to allow for the detection of the content of intracellular vesicles. By applying a constant +700 mV potential to the electrode, the vesicle membrane ruptures and opens toward the electrode tip to expose the inner materials, including catecholamines.^37^ The applied potential causes oxidation of electroactive transmitters as they diffuse to the electrode tip, and this results in amperometric spikes. A typical amperometric trace obtained by IVIEC contains a large number of spikes, and each spike comes from the oxidation of the transmitter content from a single vesicle within a single cell. Examples of IVIEC traces without or with FA incubation are shown in Figure 4A–C, and Figure 4D–F illustrates the average spike shapes for the corresponding groups. It seems that incubation with either of the two FAs gives smaller spikes, particularly ALA. To confirm the observation, several parameters of the IVIEC spikes, including *I*_max_, *t*_1/2_, *t*_rise_, and *t*_fall_, were analyzed, and the FA incubation groups were compared to the control (Table S2). For *I*_max_, a decrease is observed for ALA incubation, whereas an increase is observed for the LA group. Interestingly, for *t*_1/2_ and *t*_fall_, LA triggers a larger decrease of these two parameters than ALA does, while for SCA, the results were the opposite, with ALA giving more significant changes than LA (Table S1). The actual meaning of the parameters of IVIEC amperometric spikes remain to be understood, but they might be related to the size of the vesicle as well as the opening dynamics of the vesicle toward the electrode surface in response to the applied potential.^38,39^

**Figure 5:**
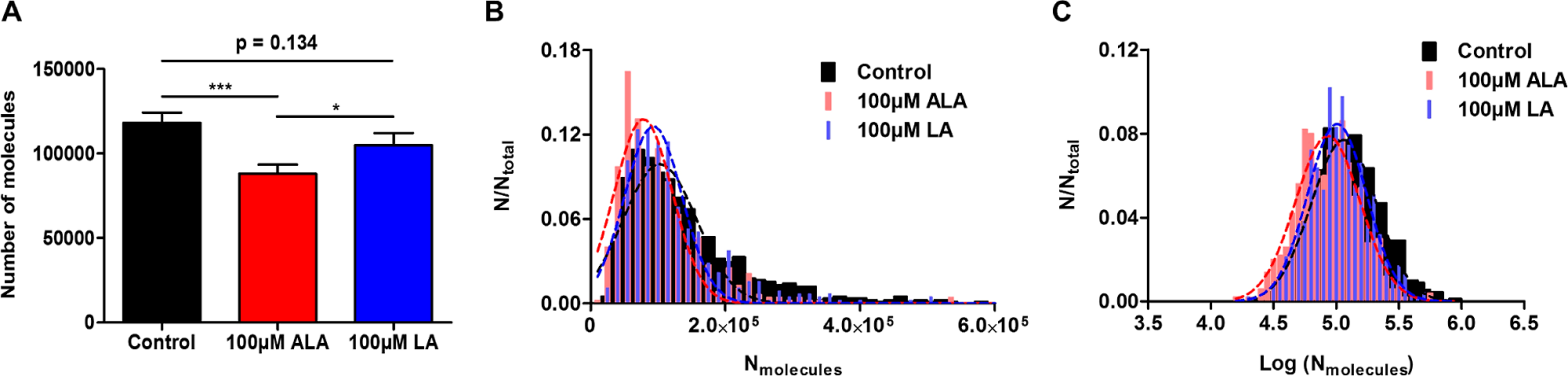
(A) Average number of molecules quantified per vesicle from PC12 cells without or with FA treatment. Error bars represent the SEM. Data sets were compared with a two-tailed Mann–Whitney rank-sum test, *p < 0.05, ***p < 0.001, and other *p* values are shown in the graph. (B) Normalized frequency histograms describing the distribution of molecules and (C) log (molecules) in vesicles from control PC12 cells (853 events from 28 cells), 24 h 100 μM ALA-treated cells (474 events from 18 cells), and 24 h 100 μM LA-treated cells (454 events from 18 cells). Bin size = 1.5 × 10^4^ molecules for (B) and 0.05 for (C). Data were fitted to a Gaussian distribution. Fits were closer to a Gaussian distribution in (C) compared to in (B).

By integrating the area under each spike from each IVIEC trace, the number of molecules stored within individual vesicles inside a single cell can be obtained, and the average amount from a population of cells is then calculated. When comparing between the control and ALA-treated cells, a significant depletion of vesicular catecholamine storage is observed as shown in Figure 5A. LA treatment does not result in significant alteration relative to the control, but the number of molecules from the LA group is obviously higher than that from the ALA group. Figure 5B,C depicts the distribution and log distribution of the number of molecules from all IVIEC events. ALA and LA treatments both result in a shift of the distribution toward the direction with smaller events. However, the change is not to the same extent as what has been seen with the distributions of SCA events (Figure 2), especially for LA treatment. The medians of the distributions for the control, ALA, and LA groups are 117,000, 89,150, and 103,000, respectively.

The fraction of release from exocytosis events can be calculated by dividing the measured release over vesicular storage. It has been found that full fusion, which results in nearly 100% release of the entire vesicular transmitter storage, is not the dominant type of vesicle fusion during exocytosis from a variety of cell types and living neurons. Instead, partial distention of the vesicle membrane results in fractional release ranging from 5 to 80% across different cell types.^33,40−44^ The fraction of release upon either ALA or LA incubation does not differ obviously from the control, with the control being 68%, ALA being 67%, and LA being 64%. Thus, it appears that although a direct effect on the fusion pore features exists, the alterations in the time of release (Figure 3) compensate for a smaller total amount in the vesicle, leading to a similar fractional release.

**Figure 6:**
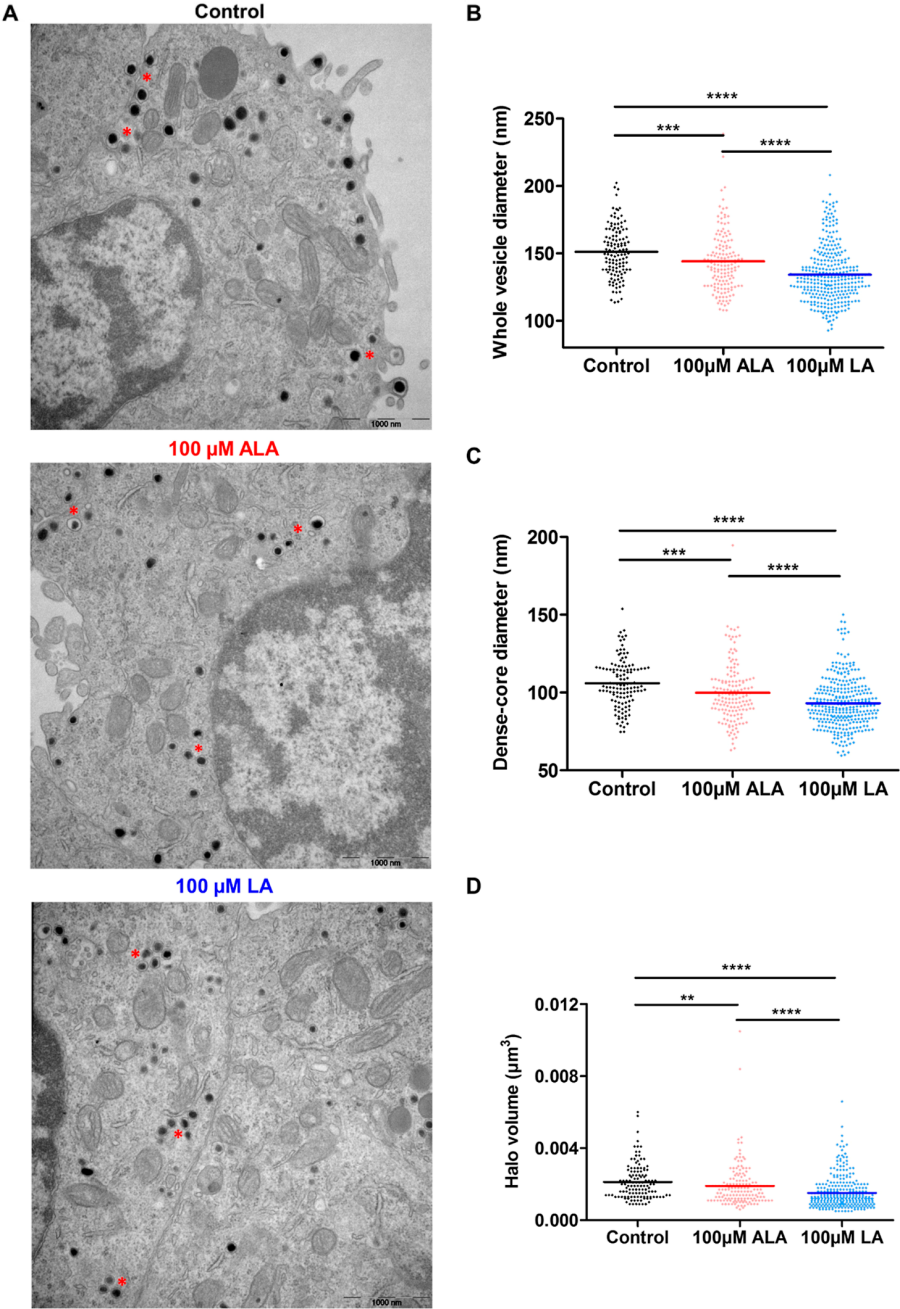
(A) TEM imaging showing vesicle and dense-core structures from control, ALA-treated, and LA-treated PC12 cells. Red asterisks indicate clusters of LDCVs. (B) Average diameters of vesicles, (C) dense cores, and (D) average volume of the vesicular halo from control (124 vesicles from 8 cells), 100 μM ALA-treated (154 vesicles from 8 cells), and 100 μM LA-treated cells (318 vesicles from 10 cells). Data sets were compared with a two-tailed Mann–Whitney rank-sum test, **p < 0.01, ***p < 0.001, and ***p < 0.0001.

One possibility that might lead to an alteration of vesicular transmitter storage is a change of vesicle size. To study this, TEM imaging was carried out to visualize and estimate the average size of vesicles after either of the two FA treatments. Only vesicles with an identifiable dense-core structure were used for the analysis. Figure 6A shows one example of the TEM images obtained for each group, and examples of clusters of LDCVs are pointed out by red asterisks. In general, vesicles shrink when treated with the ALA or LA FA, which is confirmed by the comparison of the average vesicle diameter among the three groups, shown in Figure 6B (results calculated for average vesicle diameter per cell can be found in Figure S3 in the Supporting Information). The structures of LDCVs are seen clearly in Figure 6A, consisting of an electron-dense part called the dense core and a transparent part surrounding the dense core called the halo. The dense core structure is made up of a group of chromogranin proteins that aggregate and function to bind small molecules like catecholamine transmitters.^20,45^ The halo, on the other hand, is also capable of storing some catecholamines.^46^ Manipulation of certain dense-core proteins have been previously demonstrated to alter exocytotic release as well as release dynamics.^47−49^ In addition, exocytosis of dense-core-bound neurotransmitters may exhibit different release dynamics in comparison to transmitter release directly from the halo.^50,51^ The effects on the dense-core size and volume of the halo caused by ALA or LA treatment are illustrated in Figure 6C,D, respectively. Thus, the decrease is observed not only for the size of the entire vesicle but also for both the dense-core size and the halo volume, where a proportional alteration of these three features is observed. The smaller sizes of vesicles and dense cores upon ALA treatment can be used to explain the reduced transmitter storage (Figure 5A) and faster exocytotic release dynamics (Figure 3B–D). Moreover, since the dense core and the halo are altered simultaneously by ALA, the dynamics of transmitter release from the dense core and from the halo are likely to be equally affected and thus will not have a significant influence on the total release dynamics. As for LA treatment, significant decreases are seen for the whole vesicle diameter, dense-core diameter, and the halo volume. Interestingly, when comparing between ALA and LA, the degree of decrease for LA is much larger than for ALA, which is the opposite to what has been observed for vesicular transmitter storage (Figure 5A) but the same trend as for *t*_1/2_ and *t*_fall_ from IVIEC results (Table S2). The concentrations of catecholamine in vesicles were calculated to be 104, 93, and 138 mM for control, ALA-treated, and LA-treated cells, respectively. Therefore, although smaller in size, vesicles in LA-treated cells appear to store a higher concentration of transmitters relative to ALA-treated or control cells.

### 2.3 ALA and LA Alter the Composition and Saturation Degree of Membrane Phospholipids to Influence Exocytosis

Lipids play important roles during the process of exocytosis, assisting the opening and expansion of the fusion pore via increasing membrane curvature.^9,10,52^ As FAs constitute the two tails of the membrane phospholipids, investigating the alterations in the amounts of specific membrane phospholipids caused by ALA or LA incubation is of importance for understanding their effects on exocytosis. Thus, a ToF-SIMS setup equipped with a 40 keV CO_2_ gas cluster ion beam (GCIB) was employed to analyze the lipid composition of the cell membrane upon incubation with either ALA or LA. The results illustrated in Table 1 were obtained from both positive and negative ion modes, providing a view of phospholipid species that change after the incubation. It can be observed that for ALA or LA incubation, the level of FA 18:3 or FA 18:2 increases, respectively, demonstrating direct incorporation of ALA or LA into the cell membrane. Moreover, the abundances of long-chain PUFAs that are synthesized from either ALA or LA are also elevated. To be specific, FAs 20:5, 20:4, 20:3, 22:5, 22:4, and 22:3, which are converted from ALA, have higher levels in ALA-incubated cells than in the control cells. As LA is partially converted into longer-chain PUFAs with 20 carbons, such as FA 20:4, 20:3, 20:2, and 20:1, signals of these FAs are found to be enhanced upon LA incubation. Our findings are consistent with the metabolic pathways of ALA and LA in biological systems.^53,54^ As for saturated FAs such as FA 20:0 and 22:0, both ALA and LA incubation lead to lowered signal intensities of these species. Since almost all saturated FAs have an inhibitory role in the elongation of PUFAs toward their longer-chain metabolites, it can in turn be possible that an increased level of ALA or LA inhibits the synthesis of saturated FAs.^55^

**Table 1:** Changes of the Phospholipid Composition of the PC12 Plasma Membrane after ALA or LA Incubation Analyzed Using ToF-SIMS Equipped with 40 keV (CO_2_)_6000_^+^ GCIB^a^

	100 μM ALA				100 μM LA			
	PCs	FAs	PEs	PIs	PCs	FAs	PEs	PIs
increase	PC (34:3)	FA (18:3)	PE (36:5)	PI (36:3)	PC (34:2)	FA (18:2)	PE (34:2)	PI (34:2)
	PC (36:5)	FA (20:5)	PE (36:4)	PI (38:5)	PC (34:0)	FA (20:4)	PE (36:2)	PI (36:2)
	PC (36:4)	FA (20:4)	PE (36:3)		PC (36:2)	FA (20:3)	PE (38:2)	PI (38:4)
	PC (36:3)	FA (20:3)	PE (38:5)		PC (36:0)	FA (20:2)		
	PC (38:6)	FA (22:5)	PE (38:4)		PC (38:3)	FA (20:1)		
	PC (38:5)	FA (22:4)	PE (38:3)		PC (38:2)	FA (22:4)		
	PC (38:4)	FA (22:3)				FA (22:3)		
						FA (22:2)		
decrease	PC (34:1)	FA (20:0)				FA (20:0)	PE (36:5)	PI (36:3)
	PC (34:0)	FA (22:0)				FA (22:0)	PE (36:3)	PI (38:5)
							PE (38:5)	
							PE (38:3)	

a Altered lipid species, including phosphatidylcholine (PC), FA, phosphatidylethanolamine (PE), and phosphatidylinositol (PI) are shown here. All species detected in the positive ion mode are [M + H]^+^ ions, and those detected in the negative ion mode are [M – H]^−^.

FAs are essential components forming the two tails of phospholipid molecules in human bodies.^56,57^ Several studies have shown that the intake of FAs gives rise to increased incorporation of omega-3 and omega-6 FAs into lipids of plasma or heart muscle.^58,59^ Since we observed changes in various FA species in the cell membrane due to ALA or LA treatment, the next step was to examine alterations of membrane phospholipid species. As can be seen from Table 1, cells treated with ALA have enhanced levels of phospholipid species with three to six double bonds in the structure. For instance, in the positive ion mode, the abundances of PCs 34:3, 36:3, 36:4, 38:4, 36:5, 38:5, and 38:6 increased after ALA treatment. In the negative ion mode, ALA induces enhancements of PEs 36:3, 38:3, 36:4, 38:4, 36:5, and 38:5, and PIs 36:3 and 38:5. Conversely, ALA incubation causes a reduction in phospholipid species with two double bonds, such as PIs 34:2 and 36:2. Incorporation of LA-derived FAs into the phospholipids of the cell membrane caused by LA treatment is also illustrated in Table 1, and increases are observed for phospholipid species with either two or four double bonds in the structure. For example, the levels of PCs and PEs 34:2, 36:2, and 38:2, and PIs 34:2, 36:2, and 38:4 in LA-treated cells are higher than in the control. However, the intensities of phospholipid species with three or five double bonds, including PEs 36:3, 36:5, 38:3, and 38:5 and PIs 36:3 and 38:5, appear to decrease upon LA treatment. Moreover, the PC species with three to six double bonds that are enhanced by ALA treatment are not altered by LA. Detailed information about the identified phospholipid species that are altered by ALA or LA treatment is shown as Table S3 in the Supporting Information. Taken together, ALA appears to promote the synthesis of phospholipids with three to six double bonds, while it inhibits or does not change the formation of phospholipids with two or fewer double bonds. In contrast, LA incubation enhances the synthesis of phospholipids with two or four double bonds, while it inhibits the ones with three or five double bonds. As ALA and LA compete for the same enzymes that are necessary for the synthesis of their longer-chain PUFAs, an inhibitory effect on the synthesis from LA to its longer-chain metabolites has been reported when the intake of ALA is enhanced, and the same occurs to ALA when more LA is present.^55,60^ Thus, it is reasonable that the phospholipid species (PEs and PIs) with three or five double bonds that are enhanced by ALA treatment are inhibited by LA, while the phospholipids (PIs) with two double bonds that become more abundant due to LA treatment are inhibited by ALA.

As the major component of the cell membrane, phospholipids are responsible for a variety of essential cellular processes as their function is to store energy and also determine the flexibility of the cell membrane.^61^ In this study, we found that both ALA and LA induce increased levels of unsaturated FA, PC, PE, and PI species and decrease the abundances of saturated FAs. With a higher number of double bonds present in the two FA tails of phospholipids, the tails are more bent in comparison to those without any double bonds, which makes it more difficult to tightly pack the phospholipid molecules in the membrane. As a consequence, more space is generated among the phospholipid molecules, and this promotes the flexibility as well as the permeability of the lipid bilayer membrane.^13^ Moreover, the decrease of the rigidity of membrane caused by polyunsaturated acyl chain-containing phospholipids can increase the ability of dynamin and endophilin to facilitate membrane deformation and vesiculation during endocytosis.^62,63^ As discussed earlier, since dynamin is also involved in regulating the dynamics of the fusion pore during exocytosis, an increased ability of dynamin would accelerate the activity of the exocytotic fusion pore, which explains the shorter *t*_1/2_ observed for SCA upon ALA or LA incubation (Figure 3B). When omega-3 FAs synthesized from ALA are incorporated into membrane phospholipids, this generates deformation more easily than omega-6 FAs, which are derived from LA, and this is in agreement with our results that ALA shortens *t*_1/2_ more significantly than LA does.^63^

Since cell membranes consist of two layers of phospholipid molecules, the incorporation of FAs or double bonds into either the inner or the outer leaflet of the membrane may affect exocytosis differently. In addition to that, the amount and type of the phospholipid molecule being incorporated into the plasma membrane can also influence exocytosis. When present in the plasma membrane, the shape of the phospholipid molecule, which is governed by the size of the headgroup versus the two tails, determines its location. PC has a cylindrical shape due to the similar-sized headgroup and tails and is preferentially found in flat or low-curvature regions of the membrane, e.g., more PC is present in the outer leaflet than in the inner leaflet of the membrane. PE, on the other hand, is conical shaped as the head takes less space than the tails and is therefore more prevalent in high-curvature regions of the membrane such as the inner leaflet as well as where fusion occurs during exocytosis. When incubating PC12 cells directly with PC or PE, the opposite effects on exocytotic release dynamics were reported as PC slows down vesicle fusion while PE accelerates it.^12^ The amount of release is decreased by PC incubation, but vesicles tend to be larger in size. Mass spectrometry imaging (MSI) further reveals that under the same conditions, the relative increased amount of membrane phospholipids caused by PC or PE incubation is less than 1.5%, indicating that a small alteration of certain membrane lipids is sufficient to affect exocytosis.^64^ However, when PC12 cells are treated with ALA or LA, the effect on exocytosis seems less substantial. ALA causes a minimum 50% elevation of the amount of PC, PE, or PI turnover, and the elevation reaches up to 110% for a few PI species, as shown by MSI.^8^ LA also induces a more than 40% increase of a variety of phospholipid species, but compared to ALA, the total amount of phospholipid incorporations is 40% less. Despite resulting in a significant amount of lipid turnover, the alteration of exocytosis is not as great, particularly for LA. A possible explanation for our data is that both ALA and LA increase many low- and high-curvature lipid species simultaneously (Table 1), and therefore, the outcome is more complex than when only PC or PE are used for incubation. As the phospholipids PC or PE were previously incubated for 3 days, it is highly likely that both layers of the cell membrane as well as vesicular membrane are affected.^12^ By doing short-time incubation (a few minutes) or intracellular injection, phospholipids insertion can be pinpointed to either the outer or inner layer of the cell membrane, respectively.^11,65^ In the current study, ALA or LA was incubated for 24 h, which allowed them to not only alter the cell membrane phospholipid composition but also affect the vesicular membrane lipid structure, which has a consequence of decreasing the vesicle size.

## 3 Conclusions

We studied the effects of the two essential FAs, ALA and LA, on exocytosis and the cell membrane composition to understand their potential cellular functions. PC12 cells were incubated with 100 μM ALA or LA for 24 h, and SCA and IVIEC were used to quantify the vesicular neurotransmitter release and storage, respectively. The results showed that both FAs decrease the average amount of release as well as the average amount of storage, but the decreases are more significant with ALA than with LA. Peak analysis of SCA revealed that ALA induces a faster vesicle fusion process. By imaging vesicles with TEM, we found that upon ALA or LA treatment, the size of PC12 vesicles tends to get smaller, which correlates with the lower amounts of vesicular transmitter storage measured by IVIEC. Interestingly, LA causes more reduction of vesicle size than ALA, indicating that in LA-treated cells, vesicles store a higher concentration of neurotransmitters than either ALA-treated or control cells. We further applied ToF-SIMS imaging to examine the alteration of membrane lipid species due to FAs incubations. Both FAs promote the production of longer-chain PUFAs, while they inhibit the synthesis of saturated FA species. Due to different elongation pathways, ALA and LA are synthesized to form different PUFAs, which leads to altered saturation degree of membrane phospholipids and, thus, altered membrane flexibility. We discuss how higher membrane flexibility brought by ALA incubation might give rise to increased dynamin activity, which affects the dynamics of the exocytotic fusion pore. Additionally, the amount and location of phospholipid incorporation caused by ALA or LA incubation, to various extents, also contribute to the effects observed regarding exocytosis. Our findings offer a better understanding of how exocytosis can be regulated by alteration of the membrane structure and suggest a potential pathway regarding how essential FAs protect the brain against disorders like inflammation and oxidative stress.

## 4 Methods

More details of chemicals and solutions, fabrication of nanotip electrodes, cell culture, and data processing and statistics are included in the Supporting Information. Experiments that are the most essential are presented here.

### 4.1 Electrochemical Experiments

PC12 cells were seeded on 60 mm commercial type IV collagen-coated dishes (Corning BioCoat, Fisher Scientific, Sweden) for electrochemical experiments and allowed to grow for 3 days before incubation of FAs. Right before the electrochemical experiments, medium was removed from the dish, and isotonic solution was used to wash the dish three times. Then, cells were bathed in 5 mL of isotonic solution and kept on a 37 °C heating plate during the experiment. Both SCA and IVIEC were carried out on an inverted microscope (IX71 or IX81, Olympus) inside a Faraday cage, and the potential applied to oxidize catecholamine molecules was +700 mV versus an Ag/AgCl reference electrode and was applied by an Axopatch 200B potentiostat (Molecular Devices, Sunnyvale, CA). For SCA, a nanotip electrode was placed on the top of a single cell, and a pipet filled with stimulation solution was positioned next to the cell. The pipet was connected to a microinjection device (Picospritzer II, General Valve Corporation, Fairfield, NJ), and the cell was stimulated one time for a duration of 5 s by the stimulation solution with a 20 psi pressure. For most experiments, the number of release events from each cell was greater than 20. For IVIEC, a nanotip electrode was used to pierce through the membrane of a single PC12 cell and kept inside the cell until the end of the recording. The signal outputs obtained from both SCA and IVIEC were filtered at 2 kHz and digitized at 5 kHz.

### 4.2 ToF-SIMS Sample Preparation and Analysis

PC12 cells were grown on poly-l-lysine-coated silicon wafers for 3 days and then incubated with FAs. Prior to ToF-SIMS experiments, silicon wafers were washed with warm ammonium formate solution, and excess solution on the surface was removed. The silicon wafers were then snap-frozen in isopentane and freeze-dried for ToF-SIMS analysis.

The analysis was performed using a J105-3D chemical imager ToF-SIMS instrument (Ionoptika Ltd., UK) equipped with a 40 keV GCIB with a cluster size of 6000. The ToF-SIMS imaging protocol was described in detail previously.^66^ Cells were analyzed in both positive and negative ion modes with a lateral resolution of 6 μm^2^/pixel. A primary ion current of 17 pA was used for the analysis, resulting in a primary ion density of 1 × 10^13^ ions/cm^2^. The mass range acquired for spectra was *m*/*z* 100–1000 with a mass resolution of 10,000 for *m*/*z* 772.6.

### 4.3 TEM Sample Preparation and Imaging

PC12 cells were grown in commercial type IV collagen-coated T75 flasks (Corning BioCoat, Fisher Scientific, Sweden) until confluence was reached. After incubation with FAs, cells were washed three times with warm Dulbecco’s phosphate buffered saline without calcium and magnesium and detached with TrypLE Express (Gibco, Fisher Scientific, Sweden). Cells were then centrifuged, resuspended in phosphate buffer solution, and incubated overnight at 4 °C with a modified Karnovsky fixative containing 0.01% sodium azide (BDH, UK), 1% formaldehyde, and 1.25% glutaraldehyde (Agar Scientific Ltd., UK). Afterward, cell suspensions were centrifuged at 100*g* for 10 min. Cell pellets were subsequently washed with sodium cacodylate buffer (Agar Scientific Ltd., UK) and postfixed with 1% osmium tetroxide (Agar Scientific Ltd., UK) at 4 °C for 2 h and later with 0.5% uranyl acetate at room temperature protected from light for 1 h. Samples were dehydrated in a graded series of ethanol followed by acetone and embedded in Agar 100 resin (Agar Scientific Ltd., UK). Thin sections of 70 nm were obtained with an ultramicrotome Leica EM UC 6 and placed on copper grids. Sections were counterstained with uranyl acetate and lead citrate to enhance the electron scattering properties of biological materials.^67^ TEM analysis was performed with a Leo 912AB Omega microscope at 80 kV.

## Supporting Information Available

Additional methods; average number of exocytotic release events; results of amperometric spike analysis of SCA; analysis of prespike foot parameters; results of amperometric spike analysis of IVIEC; average diameters of vesicles, dense cores, and volume of the halo from TEM imaging; and peak assignment from cells treated with either ALA or LA (PDF)
