# Supplementary material for: Omega-3 and -6 Fatty Acids Alter the Membrane Lipid Composition and Vesicle Size to Regulate Exocytosis and Storage of Catecholamines
Source: ACS Chem Neurosci. 2024 Feb 12;15(4):816–26. doi: 10.1021/acschemneuro.3c00741 (PMC10884999; doi:10.1021/acschemneuro.3c00741)
Supplement: Supplementary file 1 — cn3c00741_si_001.pdf [file cn3c00741_si_001.pdf]

SUPPORTING INFORMATION

Omega-3 and 6 Fatty Acids Alter Membrane Lipid Composition and Vesicle Size to Regulate Exocytosis and Storage of Catecholamines

Chaoyi Gu[1], Mai H. Philipsen[2], and Andrew G. Ewing[1,*]

[1] Department of Chemistry and Molecular Biology, University of Gothenburg, Medicinaregatan 19, 413 90 Gothenburg, Sweden

[2] Department of Chemistry and Chemical Engineering, Chalmers University of Technology, Kemivägen 10, 412 96 Gothenburg, Sweden

Table of Contents

Additional Methods

Chemicals and Solutions

Fabrication of Nanotip Electrodes

Cell Culture

Data Processing and Statistics

Figure S1. Average number of exocytotic release events.

Table S1. Results of amperometric spike analysis of SCA.

Figure S2. Analysis of pre-spike foot parameters.

Table S2. Results of amperometric spike analysis of IVIEC.

Figure S3. Average diameters of vesicles, dense-cores, and volume of halo from TEM imaging.

Table S3. Peak assignment from cells treated with either ALA or LA.

References

ADDITIONAL METHODS

Chemicals and Solutions

All chemicals were of analytical grade and were purchased from Merck (Sweden), otherwise their origins are stated. Cells were bathed in isotonic solution (150 mM NaCl, 5 mM KCl, 1.2 mM $MgCl_2$, 2 mM $CaCl_2$, 5 mM glucose, and 10 mM HEPES) during electrochemical measurements and stimulated for exocytosis using stimulation solution (55 mM NaCl, 100 mM KCl, 1.2 mM $MgCl_2$, 2 mM $CaCl_2$, 5 mM glucose, and 10 mM HEPES). For ToF-SIMS sample preparation, 150 mM ammonium formate solution was used to wash cell samples. All solutions used were adjusted to pH 7.4 with 3 M NaOH and filtered with a vacuum filtration system having a pore size of 0.45 µm.

Fabrication of Nanotip Electrodes

The fabrication process has been previously described.[1] Briefly, a 5 µm carbon fiber was aspirated into a borosilicate glass capillary (O.D.: 1.2 mm, I.D.: 0. 69 mm, 10 cm length, Sutter Instrument Co., Novato, CA), which was then pulled into two electrodes using a vertical pipette puller (model PE-21, Narishige, Inc., Japan). The carbon fiber outside the glass was subsequently cut to leave around a length of 100 µm and flame etched with a butane gas burner (Clas Ohlson, Sweden) to a width of 50-100 nm. The electrodes were sealed using epoxy (G A Lindberg ChemTech AB, Sweden) and baked at 100 °C overnight. All electrodes were tested in 100 µM dopamine solution by cyclic voltammetry (-0.2 V to 0.8 V vs. Ag/AgCl, 100 mV/s) and only electrodes giving stable steady-state currents were used for electrochemical measurements.

Cell Culture

PC12 cells were obtained from Lloyd Greene at Columbia University. The complete growth medium is made up of 85% RPMI-1640, 10% donor horse serum, and 5% fetal bovine serum. Cells were maintained in an incubator having 7% CO_2, 100% humidity, and a temperature of 37 °C. The flasks used for PC12 culture were commercial type IV collagen-coated T25 flasks (Corning BioCoat, Fisher Scientific, Sweden). The culture was propagated once cells were confluent which normally takes 7-9 days. FA stock solutions were made by mixing the FA with ethanol to a concentration of 300 mM.[2] For incubation of FAs, a final concentration of 100 µM ALA or LA was used to incubate PC12 cells for 24h, and the final concentration of ethanol was 0.03%.

Data Processing and Statistics

Electrochemistry data were analyzed using the Igor Pro 6.37 script written by the David Sulzer group at Columbia University. A 1 kHz Binomial sm. filter was applied to the data, and thresholds for spike and pre-spike foot detections were set to be five times and two times the standard deviation of the noise, respectively. All spikes detected by the software were manually examined and false positives were removed. Means of medians from single cells were used for all parameters for SCA and IVIEC. Graphs and statistics were done in GraphPad Prism 5 and Mann-Whitney test was applied to check significance, *: $p < 0.05$, **: $p < 0.01$, ***: $p < 0.001$, ****: $p < 0.0001$.

ToF-SIMS data were processed using Ionoptika Image Analyzer 2D software. To remove interferences from substrates, spectra from regions of interests, PC12 cells, from the ion images were extracted. The spectra were binned down to 0.1 Da and then normalized to total ion intensity of selected regions and total number of pixels.

For TEM images, ImageJ software was used to identify and select vesicle structure, including vesicle membrane and dense-core. Vesicles that had a near-spherical shape were used for size measurements and statistics, and the same criteria applied to all three groups in order to avoid potential bias. Vesicle size was calculated in volume value considering that vesicles are spherical. The halo volume is calculated by subtracting the dense-core volume from the volume of the entire vesicle. The measurement

of vesicle size in each treatment group was then compared with control and therefore, change in vesicle volume represents relative alteration of vesicle morphology.

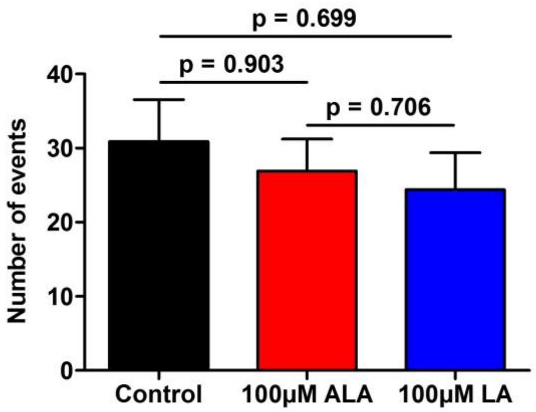

Figure S1. Average number of exocytotic release events from PC12 cells without or with FA treatment. Error bars represent SEM. Data sets were compared with a two-tailed Mann-Whitney rank-sum test and p values are indicated.

Table S1. Results of amperometric spike analysis of SCA measured from control cells (25 cells), 24 h 100 µM ALA-treated cells (21 cells), and 24 h 100 µM LA-treated cells (21 cells).

	I_{max} (pA)	$t_{1/2}$ (ms)	t_{rise} (ms)	t_{fall} (ms)
Control	15.53 ± 0.93	1.16 ± 0.07	0.33 ± 0.01	1.01 ± 0.13
100µM ALA<br>Variation relative to control	13.74 ± 0.72<br>- 12%	0.92 ± 0.05<br>- 21%**	0.29 ± 0.02<br>- 12%*	0.69 ± 0.05<br>- 32%*
100µM LA<br>Variation relative to control	14.12 ± 0.86<br>- 9%	1.06 ± 0.07<br>- 9%	0.31 ± 0.01<br>-7%	0.90 ± 0.15<br>- 10%

Data presented in the table are means of medians from each cell ± SEM. Data sets were compared with a two-tailed Mann-Whitney rank-sum test, *: $p < 0.05$, **: $p < 0.01$.

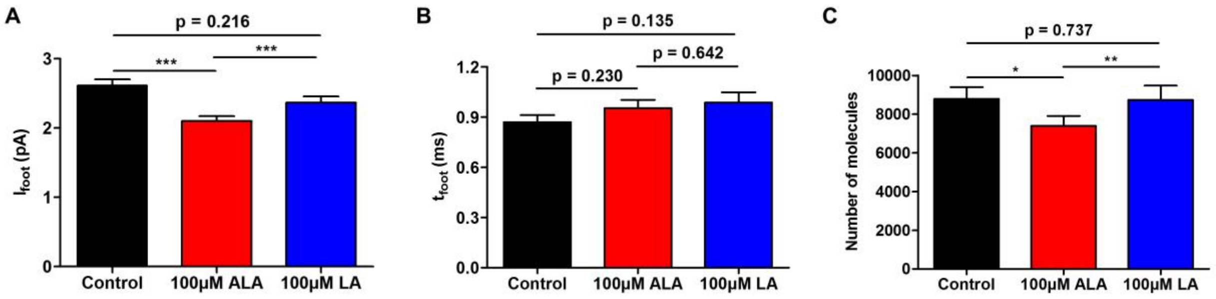

Figure S2. Plots showing the analysis of pre-spike foot parameters including (A) I_{foot} (B) t_{foot} and (C) Q_{foot} measured by SCA from control (25 cells, 244 spikes with foot in total 725 spikes), 24 h 100 µM ALA treatment (21 cells, 190 spikes with foot in total 527 spikes), and 24 h 100 µM LA treatment (21 cells, 128 spikes with foot

in total 476 spikes) cells. Data sets were compared with a two-tailed Mann-Whitney rank-sum test, *: $p < 0.05$, **: $p < 0.01$, ***: $p < 0.001$, and other p values are shown in the graph.

Table S2. Results of amperometric spike analysis of IVIEC measured from control cells (28 cells), 24 h 100 µM ALA-treated cells (18 cells), and 24 h 100 µM LA-treated cells (18 cells).

	I_{max} (pA)	$t_{1/2}$ (ms)	t_{rise} (ms)	t_{fall} (ms)
Control	19.53 ± 1.45	1.25 ± 0.06	0.30 ± 0.01	1.05 ± 0.07
100µM ALA<br>Variation relative to control	16.49 ± 0.88<br>- 16%	1.16 ± 0.09<br>- 7%	0.34 ± 0.03<br>+ 10%	0.93 ± 0.08<br>- 12%
100µM LA<br>Variation relative to control	21.72 ± 1.94<br>+ 11%	1.09 ± 0.04<br>- 12%<br>(p = 0.085)	0.31 ± 0.02<br>+ 1%	0.87 ± 0.06<br>- 18%<br>(p = 0.108)

Data presented in the table are means of medians from each cell ± SEM. Data sets were compared with a two-tailed Mann-Whitney rank-sum test and some p values are shown in the table.

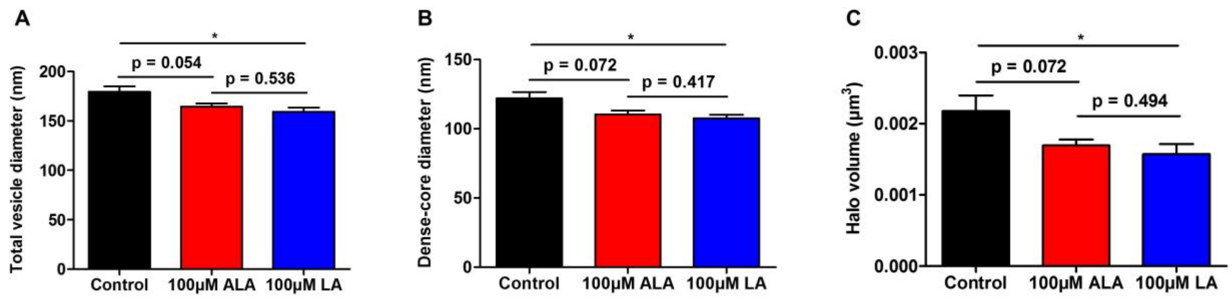

Figure S3. (A) Average diameters of vesicles, (B) dense-cores, and (C) average volume of vesicular halo per cell from control (8 cells), 100 µM ALA-treated (8 cells), and 100 µM LA-treated cells (10 cells). Error bars represent SEM.

Table S3. Peak assignment from cells treated with either ALA or LA.

Positive ion mode $[M+Na/K]^+$		Negative ion mode $[M-H]^-$	
Assignment	Measured m/z	Assignment	Measured m/z
PC (34:2) + Na	780.6	FA (18:2)	279.2
PC (34:3) + Na	778.5	FA (18:3)	277.2
PC (36:2) + K	824.6	FA (20:0)	311.3
PC (36:3) + K	822.5	FA (20:1)	309.3
PC (36:4) + K	820.5	FA (20:2)	307.3
PC (36:5) + K	818.5	FA (20:3)	305.2

PC (38:2) + Na	836.5	FA (20:4)	303.2
PC (38:3) + Na	834.5	FA (20:5)	301.2
PC (38:4) + Na	832.5	FA (22:0)	339.3
PC (38:5) + Na	830.5	FA (22:3)	333.3
PC (38:6) + Na	828.5	FA (22:4)	331.3
		FA (22:5)	329.2
		PE (34:2)	714.5
		PE (36:2)	742.5
		PE (36:3)	740.5
		PE (36:4)	738.5
		PE (36:5)	736.5
		PE (38:2)	770.6
		PE (38:3)	768.6
		PE (38:4)	766.5
		PE (38:5)	764.5
		PI (34:2)	833.5
		PI (36:2)	861.6
		PI (36:3)	859.5
		PI (38:4)	885.6
		PI (38:5)	883.5

REFERENCES

1. Li, X., Majdi, S., Dunevall, J., Fathali, H., and Ewing, A. G. (2015) Quantitative measurement of transmitters in individual vesicles in the cytoplasm of single cells with nanotip electrodes, *Angew Chem Int Ed 54*, 11978-11982.

2. Philipsen, M. H., Samfors, S., Malmberg, P., and Ewing, A. G. (2018) Relative quantification of deuterated omega-3 and -6 fatty acids and their lipid turnover in PC12 cell membranes using TOF-SIMS, *J Lipid Res 59*, 2098-2107.
